# Magnetic Hyperthermia and Radiation Therapy: Radiobiological Principles and Current Practice †

**DOI:** 10.3390/nano8060401

**Published:** 2018-06-03

**Authors:** Spiridon V. Spirou, Martina Basini, Alessandro Lascialfari, Claudio Sangregorio, Claudia Innocenti

**Affiliations:** 1 Department of Radiology, Sismanoglio General Hospital of Attica, Sismanogliou 1, Marousi 15126, Greece; 2 Università degli Studi di Milano, Dipartimento di Fisica, Via Celoria 16, 20133 Milano, Italy; martina.basini@gmail.com (M.B.); alessandro.lascialfari@unimi.it (A.L.); 3 ICCOM-CNR via Madonna del Piano 10, 50019 Sesto Fiorentino, Italy; csangregorio@iccom.cnr.it; 4 INSTM and Dept. Of Chemistry “U. Schiff”, University of Florence, via della Lastruccia 3, 50019 Sesto Fiorentino, Italy; claudia.innocenti@unifi.it

**Keywords:** hyperthermia, radiation therapy, magnetic nanoparticles, magnetic fluid hyperthermia, nanomedicine, cancer therapy

## Abstract

Hyperthermia, though by itself generally non-curative for cancer, can significantly increase the efficacy of radiation therapy, as demonstrated by in vitro, in vivo, and clinical results. Its limited use in the clinic is mainly due to various practical implementation difficulties, the most important being how to adequately heat the tumor, especially deep-seated ones. In this work, we first review the effects of hyperthermia on tissue, the limitations of radiation therapy and the radiobiological rationale for combining the two treatment modalities. Subsequently, we review the theory and evidence for magnetic hyperthermia that is based on magnetic nanoparticles, its advantages compared with other methods of hyperthermia, and how it can be used to overcome the problems associated with traditional techniques of hyperthermia.

## 1. Introduction

“Those diseases that medicines do not cure, are cured by the knife. Those that the knife does not cure, are cured by fire. Those that fire does not cure, must be considered incurable.” [1]. Thus, Hippocrates of Cos (c. 460–370 B.C.), the father of medicine, described the role of heat in medicine (Aphorisms VII, 87).

In oncology, the first reference to using heat as a treatment modality appears in the Edwin Smith Surgical Papyrus [2]. The Papyrus dates from c. 1700 B.C. but is believed to be a copy of an earlier text, c. 3000-2500 B.C. In Case 39, which discusses breast tumors, the physician is instructed to “burn” (cauterize) the disease using a “fire-stick” or “fire-drill”.

In 1866 the German physician W. Busch reported the case of a sarcoma patient whose tumor regressed due to high fever caused by infection with erysipelas [3]. Similar observations by others led to the use of fever-inducing toxins derived from erysipelas-causing bacteria. The most noted among these efforts was led by New York surgeon W.B. Coley [4].

In 1910 Müller suggested the combination of hyperthermia and radiation therapy [5]. In the ensuing decades, investigations were hampered by the lack of equipment and understanding of cell biology. In the 1960s and 1970s, interest increased significantly, leading to seminal publications on the cellular effects of hyperthermia and its synergy with chemotherapy or radiation therapy [6,7,8,9,10,11,12,13,14,15,16,17,18].

These studies showed that, although hyperthermia alone can have deleterious effects on cells, including sometimes killing them, its real strength comes when it is used as an adjunct to chemotherapy or radiation therapy. In this work we focus on the latter, namely, the synergy between hyperthermia and radiation therapy. We review the effects of hyperthermia, the limitations of radiation therapy, the radiobiological evidence for combining the two treatment modalities and the special characteristics of magnetic hyperthermia.

## 2. Hyperthermia

The term hyperthermia (HT) generally refers to the heating of cells and tissues, for one hour or more, to a temperature between 40 and 45 °C [19,20,21,22], although the exact limits differ between authors. Sometimes the terms mild hyperthermia or just hyperthermia is used for temperatures up to 42 °C and extreme hyperthermia for higher temperatures [23]. At those temperatures, heat alone may cause protein denaturation [24], damage to the cytoskeleton [25,26], impairment of certain DNA repair processes [27,28,29,30,31,32], changes in cell membrane permeability [33,34], and stimulation of the immune system [35,36,37,38].

In addition, hyperthermia below 42 °C modulates the microenvironment and increases blood flow to the tumor, as well as vascular permeability, thus potentially improving the supply of oxygen and nutrients to tumor cells. At the same time, the increased perfusion will act as a coolant, carrying the applied heat away, unless measures are taken to counter this effect. The increase in perfusion begins a few minutes after heating and may last for several hours. Above 42 °C the tumor vessels may collapse, trapping the applied heat, and resulting in necrosis or apoptosis [6,20,22,39,40,41,42,43,44,45,46,47,48,49,50]. At this temperature, HT may also cause coagulation, especially with perfusion or whole-body HT techniques. This will, in turn, induce hypoxia, thus contributing to tumor radioresistance [22,51,52,53,54,55,56].

Moderate HT also reduces oxygen consumption by turning cell metabolism towards the glycolytic pathway and by reducing respiration in sub-lethally heated cells [57,58]. This increases the oxygen that is available and reduces hypoxia.

These effects are generally more pronounced in tumor cells than normal tissue, although this is not due to the intrinsic thermosensitivity of the cells but, rather, the acidic microenvironment of tumor cells. The magnitude of these effects in different tissues varies and, by themselves, they cause little to moderate cytotoxicity; however, when the cell suffers additional damage by chemotherapy or radiation therapy, cytotoxicity increases rapidly, much more than the simple addition of each effect [21,22,59,60,61].

Experiments in vitro showed that there is a clear breakpoint in the response of cells to heat [6,62]. At temperatures below the breakpoint, the slope of the survival curve becomes shallower as heat shock proteins are expressed to protect the cell from further damage, thus inducing thermotolerance. At temperatures above the breakpoint, the rate of cell killing doubles with each increase by 1 °C.

The breakpoint occurs at approximately 43 °C for rodent and 43.5 °C for human cell lines. Since the earlier experiments were conducted on rodent cells, the temperature of 43 °C was chosen to be the standard reference temperature for the comparison of different heating schemes (combination of temperature and time):

(1) $$CEM\;43\;{}^{\circ}C=\int_{0}^{t}R^{\left(43-T\right)}\cdot dt\;where\;R=\left\{ \begin{array}{ll} 0.5 & for\;T>43\;{}^{\circ}C\\ 0.25 & for\;T<43\;{}^{\circ}C \end{array}\right.$$

where *CEM* 43 °C is the cumulative equivalent minutes at 43 °C, *t* is the duration of treatment, and *T* is the heating temperature [63].

Since the actual temperature distribution in the tumor may vary significantly and treatment success depends on the minimum temperature achieved, the quantity usually quoted is *CEM* 43 °C *T*90, which is the *CEM* 43 °C to 90% of the tissue. Clinical studies have shown that it correlates well with clinical outcomes [64,65,66,67,68,69,70,71].

It should not be forgotten, however, that *CEM* 43 °C is nothing more than a mathematical expression to convert and compare different heating schemes. It does not take into account biological effects or the interaction and synergy between hyperthermia and radiation therapy or chemotherapy [72,73].

## 3. Radiation Therapy

In contrast to HT, radiation therapy (RT) has been a standard treatment modality for cancer ever since the discovery of X-rays by Roentgen. It is currently administered to more than half of all cancer patients, either alone or in combination with surgery and/or chemotherapy [74]. Its success or failure is ultimately defined by tumor radioresistance, which determines how much dose should be delivered, and normal tissue toxicity, which determines how much dose can be delivered.

Normal tissue toxicity can be mitigated by improvements in technology, such as intensity-modulated radiation therapy [75,76,77,78,79], volumetric modulated radiation therapy [80,81,82], image-guided radiation therapy [83,84,85], stereotactic body RT [86,87,88,89], proton therapy [90,91,92] etc., that more tightly focus and conform the high-dose region to the tumor, as well as various techniques that try to spare normal tissue, such as fractionation, irradiating from multiple angles etc.

Ionizing radiation primarily targets the DNA molecule either directly, by breaking bonds, or indirectly, by creating reactive oxygen species (ROS) or free radicals. Since most of the cell is made up of water, these are usually hydroxyl ions (OH^−^). For photons, which is the most common modality used in the clinic, the indirect action accounts for about 70% of the total damage to the DNA [93].

ROS react with the DNA molecule, breaking a bond and forming a new compound. This new compound is highly unstable and, if left alone, the reaction is often reversed, the DNA bond break is restored, and the ROS finds a hydrogen atom to become neutral. However, if oxygen is present, the reaction proceeds further to form a new stable compound, thus making the bond break permanent. Single-strand breaks are repairable whereas double-strand breaks are sometimes not, eventually leading to cell death [94,95,96,97].

Tumor radioresistance stems primarily from hypoxic regions. As tumors grow more rapidly than the surrounding tissues can support, they develop their own vasculature. However, this vasculature is morphologically and functionally primitive and is unable to properly supply oxygen and nutrients to every cell in the tumor [98]. This creates hypoxic regions in the tumor, which are radio-resistant, since oxygen is not available for the free radicals to inflict permanent damage to the DNA. In some cases, hypoxic cells may require as much as triple the radiation dose as oxic cells in order to achieve the same effect [99].

An alternative approach to increasing the radiation dose is to use radiosensitizing agents in the tumor. HT is one such agent that increases the efficacy of RT. This does not only mean a higher probability of eradicating the tumor, but also lower normal tissue toxicity as lower doses may be sufficient.

## 4. Synergy between HT and RT

The synergy and complementarity between HT and RT have been described in several reviews [21,22,100,101,102,103,104] and are briefly summarized here.

(a) Hypoxia: Hypoxic cells are radioresistant but heat-sensitive [8,105,106,107,108].
(b) Cell cycle: Cells that are undergoing mitosis are radiosensitive whereas cells in the S phase are radio-resistant. The reason for this is that cells in the S phase go into an arrest mode, stopping further progress in the cycle until DNA sublethal damage has been repaired. In contrast to radiation, cells in the S phase are the most sensitive to heat [12,18,109,110,111].
(c) Tumor Microenvironment: In addition to a lack of nutrients, the tumor microenvironment is characterized by a low extracellular pH. This makes tumor cells more radio-resistant but heat-sensitive [7,59,112,113,114,115,116,117,118].
(d) Heat dissipation: When tissue is externally heated, the normal vasculature expands and blood flow is increased in order to carry the heat away. In tumors, however, the morphologically and functionally primitive vasculature is unable to do this, so that the tumor is selectively heated vis-a-vis normal tissue [20,43,46,48,119].
(e) Heat concentration in the tumor: As described previously, within the tumor there is uneven flow of blood resulting in hypoxic regions. Thus, when these regions are heated, the vasculature is unable to dissipate the heat, so that heat is trapped in hypoxic regions [43,46,48,119].

Clinical studies, including Phase II and Phase III trials, have supported the above in vitro and pre-clinical results and have shown that HT can significantly improve outcomes when combined with RT [19,120,121,122,123,124,125,126,127,128,129,130,131]. For example, complete response increased from 38.1% with RT alone to 60.2% with HT and RT in locally recurrent breast cancer [122], from 39.6% to 62.5% in head and neck cancers [123], and from 48–58% to 72–83% in primary cervical cancer [125].

## 5. Clinical Implementation

Despite the evidence presented previously, the different approaches to HT (local, regional and whole-body) and the variety of heating devices (microwave, radiofrequency, ultrasound, infrared, blankets, implants, perfusion techniques etc.), HT is not routinely used in the clinic, with the exception of the Netherlands. In the Tilburg and Academic Medical Center clinics, HT is used in recurrent breast cancer, in order to reduce the radiation dose, and in cervical cancer for patients who are allergic to cisplatin.

A key problem is the difficulty of adequately heating the tumor, especially deep-seated ones [22,119]. This was vividly demonstrated in a Phase III Radiation Therapy Oncology Group (RTOG) study that compared interstitial radiotherapy with and without hyperthermia. The study found no difference when HT was included in the treatment. However, when criteria for the adequacy of HT were applied, it turned out that only one patient had actually fulfilled them [132].

Further, as in RT, it is important not only to deliver the desired heat or dose to the tumor, but also to focus it to the tumor and spare surrounding normal tissue. This is especially an issue when heat is delivered externally, since conduction and dissipation may play an important role.

At present, the optimum temperature distribution is unknown and, therefore, researchers and clinicians are aiming at a steady, homogeneous temperature. This mirrors the traditional approach to RT. However, the past 20 years have demonstrated that non-traditional fractionation schemes (e.g., hypofractionated) and non-uniform radiation doses may be advantageous, as the radiosensitivity of the tumor varies spatially and parts of it may require a higher dose for treatment (“dose painting”). Hence, one should be open to the possibility that non-uniform temperature distributions and heating schemes may also be advantageous.

Lastly, another complication is the uncertainty in measuring the temperature distribution in the tumor and surrounding normal tissue. In most cases, the temperature is measured using probes, which are invasive and practically limited to only a few points. Magnetic resonance imaging can be used to get a map of the 3D temperature distribution but, unless a dedicated unit is available, scheduling issues are bound to arise [133]. Other methods, such as infrared imaging, are 2D and indirect, since they measure the surface temperature.

Magnetic nanoparticles and magnetic HT have the potential to overcome these problems. This is further discussed in Section 6 and Section 7.

## 6. Magnetic Hyperthermia

Magnetic hyperthermia (MHT) generally refers to the heat released by specific inductive mediators during exposure to an alternating magnetic field (AMF) of appropriate frequency and amplitude. Although HT is also induced by the electrical component of the EM radiation, the main advantage of MHT is its selectivity due to the natural transparency of the human body, the relative permeability of which is nearly 1, to the magnetic field. The first approaches for exploiting this property involved electro-conductive or ferromagnetic components, such as needles or seeds of micrometric size, implanted inside the tumor tissue by surgery.

MHT received a large impulse in recent years when colloidal dispersions of magnetic nanoparticles (MNP) were proposed as novel heat mediators able, in principle, to provide the opportunity of tumor targeting through blood circulation or direct local injection, without surgery, and to release heat in situ, once the external AMF is activated [20,134].

This procedure thus leads to focus-generated heat on the affected cells. The huge growth, in recent years, of research on nanostructured materials suitable for this purpose led to the birth of a novel, specific kind of HT treatment, denoted as magnetic fluid hyperthermia (MFH). Fluids are indeed used as stable colloidal suspensions of MNPs in liquid media, such as water or hydrocarbon fluids [135]. The large number and overall surface of MNPs in the suspensions assure excellent power absorption and release capabilities, which make them particularly suitable for contactless, selective interstitial heating of tumors [136].

In the last 20 years, various MNPs with different properties and morphological structures have been applied for MHT therapy [137]. Among them, iron oxide nanoparticles have been recognized as prominent candidates for HT, because they are “naturally” biocompatible (iron can be recycled through the metabolic pathways) and can be easily synthesized at the nanoscale size [138]. In particular, spinel ferrite magnetite (Fe_3_O_4_) and maghemite (γ-Fe_2_O_3_), or mixed ferrites (general formula: M_x_Fe_3−x_O_4_ (M = divalent metallic cations)) with high oxidative stability are extensively studied because of their biological compatibility and superior magnetic properties [139].

### 6.1. Basics and limitations of MFH

The absorption of the energy of an external EM field by a material and its consequent heating strongly depend on the material properties involved in the response to the electric and magnetic components of the field, namely, the electric permittivity, ε, and the magnetic permeability μ, respectively. Since both depend on the applied frequency, *f*, and field amplitude, *H*, the external field parameters must be carefully chosen to optimize the heating performance of the MNPs against cancer cells, while preserving healthy tissues and patient comfort during treatment. The electric permittivity of biological tissue is high enough to produce undesirable induced currents in the tissues, which can result in non-selective heating and uncontrollable “hot spots”. A first challenge is thus to reduce the electrical component of the EM field by properly designing the emitter. Eddy currents, however, can occur in the presence of the magnetic component only, and the power released can be estimated to be proportional to the square of (*H • f • D*) where *D* is the induced current loop diameter.

The small size of MNPs represents the first important constraint to the development of eddy currents, as *D* cannot exceed the particle characteristic length. The frequency is recommended to be limited in the interval 50 kHz < *f* < 1 MHz, since other deleterious physiological responses can occur with increasing frequency, like muscle stimulation (peripheral and skeletal), cardiac stimulation, and arrhythmia [140,141]. The limitations on the amplitude of the applied field (*H* < 15 kA/m) can be roughly estimated by considering the product *H • f*, which represents the dependence of the eddy current dissipation on the external parameters. Brezovich followed this approach by establishing experimentally the maximum value of *H • f* to be equal to 4.85 × 10^8^ A m^−1^ s^−1^, in order to guarantee the safety and comfort of the patients during clinical application. Despite its empirical basis and the decades that have passed from its establishment, this value is still referred to as the human tolerance threshold to the external field application in MFH [142,143]. In certain cases, different values of *H • f* have been suggested. For instance, the increase of one order of magnitude of the exploitable *H • f* product has been proposed when small and not-fragile body regions are exposed [144]. An extended discussion on this subject, involving the scientific and clinical communities, should be encouraged to provide a better analysis and more exhaustive indications on the application limits to support and orient the material research toward the realization of devices exploitable in clinics.

Once the appropriate range of fields and frequencies has been established, the magnetic properties of the material employed must be considered for managing the heating process. The candidate materials for MHT are ferromagnetic (or ferrimagnetic) nanoparticles (FM), which can assume a superparamagnetic behavior (SPM) at room temperature when their size is reduced below the critical value for the formation of magnetic domains (single domain MNPs). In this case, the sample magnetization is not persistent but relaxes to zero with a characteristic time, which depends on the ratio between the magnetic anisotropy energy of the system, *E_a_*, and the thermal energy (Néel relaxation time, *τ_N_*). The choice of MNP size and field frequency range allows the exclusion of heating processes related to eddy currents, as already mentioned, and to ferromagnetic resonance, which occurs in the GHz range. The heating mechanism to be considered is thus due to the magnetic energy losses of the material, occurring when the magnetization is not able to follow synchronously the external field orientation (relaxation losses) or presenting a hysteretic behavior (hysteresis losses).

The first case is characteristic of single domain MNPs in the superparamagnetic regime and its efficiency strongly depends on the frequency, as it is related to the product between the effective relaxation time of the system, $\tau_{eff}$, and the frequency of the external field.

(2) $$\frac{1}{\tau_{eff}}=\frac{1}{\tau_{N}}+\frac{1}{\tau_{B}}$$

where $\tau_{B}$ is the characteristic Brown relaxation time.

Indeed, for values of the field amplitude, *H*, low enough to approximate the magnetization with a linear function of *H* (Linear Response Theory), the dissipated power is given by [145]:

(3) $$P_{spm}=\mu_{0}\pi \chi_{0}H\frac{2\pi f\tau_{eff}}{1+{\left(2\pi f\tau_{eff}\right)}^{2}}$$

where $\chi_{0}$ is the isothermal susceptibility.

When the external frequency is much higher than $1/\tau_{eff}$ or when the LRT cannot be applied or when the FM system presents a multidomain structure, the absorbed energy is generically quantified by the area of the hysteresis loop, *A*, and the dissipated power by the product *A • f*.

Since the recommended range of magnetic fields for MHT is restricted, the power dissipated by hysteresis losses is strongly reduced, because only minor loops can be exploited. For low fields, the use of SPM nanoparticles is thus generally preferred, even though the cross-relationship of the external parameters with the properties of the material is complex and a careful analysis or numerical simulations should be carried out to choose the appropriate combination to achieve the maximum efficiency with the lowest MNP dose.

Besides the modeling of the magnetic behavior driving the heat release process, the hyperthermic capability of the material can be directly assessed by evaluating the specific absorption rate (SAR) or specific loss power (SLP) by calorimetric measurements, since, for an adiabatic system, the power needed for increasing the temperature of Δ*T* in the time interval Δ*t* is, by definition, the specific heat of the sample, *c*, multiplied by Δ*T*/Δ*t*. Extended to a system with *n* components (generally, solvent and MNPs), the total absorbed power per unit of mass becomes:

(4) $$SAR=\frac{\sum_{i=1}^{n}m_{i}c_{i}}{m_{MNP}}\cdot \frac{\Delta T}{\Delta t}$$

where *m_i_* and *c_i_* are the mass and the specific heat of the *i*-species. In recent years, due to important developments in materials research to improve the performance of MFH mediators, several commercial instrumentations for the calorimetric evaluation of SAR have appeared on the market (Nanotherm^®^, Biomagnetics^®^). Neither these nor the experimental set-ups built in research laboratories (except from the one in Zaragoza [146]), however, provide true adiabatic conditions for the measurements. The most common method to reduce the effect of heat dissipation is to evaluate the SAR from the initial slope of the temperature kinetics curve. Alternatively, the whole *T*(*t*) curve can be fitted to specific functions, one of the most commonly used being the Box Lucas one, that involves unknown dissipation parameters [147,148].

Besides the accuracy of the model used for the experimental estimate of the SAR, other important items make the evaluation of the SAR a critical issue. As mentioned before, the energy absorbed during the AMF exposition is intrinsically dependent on the external field parameters and, in most cases, cannot be easily rescaled to exclude or reduce this dependence. Therefore, the various numbers of SAR values reported in the literature are hardly comparable to each other, as they refer to different fields and frequencies, which, in many cases, are well above the Brezovich threshold (some examples in the following paragraph). Except for some isolated attempts to reduce the external field contribution influence by defining new quantities to identify the hyperthermic efficacy (see, for instance, the “Intrinsic Loss Power” (ILP) [149,150]), up to now, no serious effort has been made to find a commonly accepted standardization of the SAR evaluation. 

In addition to the above physical considerations, the main biological issue is the route of administration. Direct or intratumoral administration guarantees that the MNPs are located in the tumor, but is an invasive procedure that runs the risk of spreading the disease, and may not always be an option, especially for deep-seated tumors. Systemic administration is easier, achieves higher internalization, but results in a low accumulation rate in the tumor. This is due to capture and elimination by phagocytes and the reticuloendothelial system [151,152]. Further, as the MNPs tend to accumulate in the liver, they could cause liver damage after treatment with HT [153,154].

### 6.2. Clinical Studies

Animal studies on mouse mammary carcinoma, glioblastoma, and prostate cancer have demonstrated the feasibility and efficacy of MFH, as well as a very low clearance rate of the MNPs from tumors, allowing for serial heat treatments after a single magnetic fluid injection [155,156,157,158,159,160,161].

Clinical studies of MFH were begun by Jordan et al. (http://www.magforce.de/en/home.html) [162,163,164,165] who presented the prototype of a whole-body magnetic field applicator (MFH^®^300F) in 2010 [166]. The study was carried out at the MagForce Charité Hospital in Berlin, where clinical trials of MFH on patients affected by glioblastoma multiforme, prostate and pancreas tumors were performed. The magnetic field applicator (NanoActivator^®^) is large enough for human patients and operates with alternating magnetic field of 2–15 kA/m amplitude and 100 kHz frequency, thus demonstrating the feasibility of the therapy from a technical point of view.

Significant benefits of MFH in treating the tumor, however, were reported only in combination with radiotherapy and using 12 nm magnetite MNPs coated with aminosilane as heat mediators, directly injected in the tumor mass at high doses (ca. 30 mg/cm^3^ of tissue).

In 2005, Johannsen et al. performed a pilot study and presented the first clinical application of interstitial MFH using MNPs for treatment of prostate cancer [167]. A MNP suspension was injected into the prostate, under ultrasound and fluoroscopy guidance, in a patient with previously irradiated and locally recurrent prostate carcinoma. The study showed that MFH was feasible and well-tolerated. Thereafter, several clinical trials on patients with glioblastoma multiforme, prostate, esophagus, and liver cancers were performed to investigate the potential role of MFH [165,168,169,170]. Depending on the treated region, the strength of the magnetic field ranged from 2 to 18 KA/m.

Wust et al. [171] performed a clinical evaluation of the feasibility, tolerance and temperatures achieved by a magnetofluid (MFL 082AS) and a whole-body magnetic field applicator (MFH^®^300F). The study was conducted on 22 patients with various types of recurrent tumors (sarcoma, rectal cancer, cancer cervix, ovarian cancer, and prostate cancer) that had been previously heavily treated. Different field amplitudes were applied, depending on the pathology (3–6 kA/m in the pelvis, up to 7.5 kA/m for neck and thorax and >10 kA/m for the head). The SAR achieved was 60–380 W/kg in the target and, on the average, 86% of the tumor was heated to more than 40 °C, although there was wide variation. Coverage with >42 °C was unsatisfactory; however, the authors estimated that it could increase to 98% with a moderate increase of the magnetic field. The procedure was well-tolerated. Most patients reported subjective feelings of heat or heat stress, but that did not impact the treatment. Subacute toxicities at one year after treatment were moderate.

Maier-Hauff et al. [164] investigated the efficacy and tolerability of MFH combined with RT on recurrent glioblastoma multiforme. Fourteen patients were recruited in a phase I trial. A suspension of iron oxide MNPs was injected in the tumor and its spatial distribution was monitored by computed tomography. The patients received 4–10 (median 6) sessions of thermotherapy and 16–70 Gy (median 30) of radiotherapy dose at 2 Gy per fraction. The median maximum intratumoral temperature was 44.6 °C (42.4–49.5 °C), T90, the temperature exceeded by 90% of the tumor, was 40.5 °C and CEM 43 °C T90 was 7.7 min. The combination treatment was tolerated well by all patients with minor or no complications or side effects. Although not a primary endpoint of the study, an increase in the median survival rate of the patients is expected.

This was followed by a phase II study, in which the primary endpoint was patient survival [165]. Approximately 5 mL of 12 nm Fe_3_O_4_ nanoparticles coated with aminosilane were directly injected into recurrent glioblastomas prior to twice weekly HT treatment in a 100 kHz alternating magnetic field, concurrently with 30 Gy of external beam radiation at 2 Gy/fraction (five times/week). The median overall survival of 13.4 months (10.6–16.2 months) reported in 59 patients was substantially longer than the typical six months median survival noted in such patients [172,173]. The procedure was very well tolerated and post-mortem analyses noted that nanoparticles were confined to areas of tumor necrosis and localized within macrophages rather than cancer cells [174].

Recently, Matsumine et al. [175] were able to apply the HT treatment to metastatic bone tumors, even though bone metastases have several special properties which discourage oncologists from using HT therapeutic strategies.

Despite the improvements described above, current clinical applications are still far from the great potentiality of MFH therapy discussed before. An important limitation to the development of clinical routine treatments is represented by the scarce power loss available in the target tissue. As evaluated by Hergt et al. [144], indeed, in order to enhance the temperature of a 3 mm tumor (i.e., the smallest size diagnosed as metastasis) by 5 K, a huge amount of power is required, which can be obtained by a concentration of 1 mg/cm^3^ of magnetic mediators with SAR of 10 kW/g, a value which is far from those currently available using field parameters within the Brezovich limit. Even though for larger tumors the power needed for obtaining the same temperature is reduced as the square of the size, the improvement of the hyperthermic properties of MNP mediators is regarded as the main strategy to reach the clinical requirements, leading to intense research activity and, consequently, a large number of publications on the synthesis and characterization of MFH heat mediators with advanced hyperthermic performances. The choice of the investigated material, however, is restricted by the demand for biocompatibility, non-toxicity, and chemical stability in the physiological environment, appropriate circulation time in blood and, finally, harmless biodegradability. The majority of the investigations, particularly in most of the early literature have thus focused on magnetic iron oxides Fe_3_O_4_ (magnetite) and γ-Fe_2_O_3_ (maghemite) [176,177], which have been proven to be well-tolerated by the human body, as they can be recycled in the natural metabolic process.

The typical SAR values for magnetite and maghemite MNPs reported in the literature, are very spread in the interval 10–200 W/g, in part due to the different field parameter values used. Notable exceptions are represented by 35 nm bacterial magnetosome 960 W/g at 410 kHz and 10 kA/m [178] and 16 nm γ-Fe_2_O_3_ MNPs whose SAR has been recorded as high as 1650 W/g at 700 kHz and 24.8 kA/m (drastically reduced to 300 W/g at 11 kA/m [179]). Only recently, several systems alternatives to spinel iron oxides have been investigated, mainly focusing on the enhancement of SAR by increasing the magnetic moment. The materials investigated include pure metal such as Co [180], metal alloys such as FePt [181,182], core-shell systems such as Fe_3_O_4_@Fe, Au@Fe_3_O_4_, and Au@Co [183,184], and doped ferrites [185]. Among the highest values reported, SAR = 1700 W/g was measured for FeCo MNPs [182] at *f* = 300 kHz and *H* = 52.8 kA/m. Extraordinary high values of SAR were also reported for exchange coupled core-shell nanostructures comprising hard and soft mixed ferrites, the best example being Zn_0.4_Co_0.6_Fe_2_O_4_@Zn_0.4_Mn_0.6_Fe_2_O_4_, which exhibited a SAR of 3886 W/g [186]. Note that in these last cases the product *H • f* greatly exceeds the Brezovich threshold (36 and 33 times, respectively), confirming the urgent need for clarifying this point to progress toward systems really exploitable in clinics, as previously mentioned.

It has been shown both theoretically and experimentally that the maximum hyperthermic efficacy for iron oxides is attained for size in the 15–20 nm range for magnetite [179], and 20–25 nm for maghemite [139,187]. The use of magnetic materials with large magnetic anisotropy has proved to be an excellent strategy to reduce this size while keeping high SAR values. One of the most noticeable examples is partial or total substitution of the divalent iron of the spinel ferrite with divalent cobalt (doped Co_x_Fe_3−x_O_4_ ferrites), resulting in SAR values 3–5 times larger than those of iron oxide of similar size [188,189,190].

Recently, the *in situ* reduction method was applied for shape-controlled of Fe_3_O_4_ with Ag coating into the core-shell (Fe_3_O_4_@Ag) or heterodimer (Fe_3_O_4_-Ag) structures. Both coated Fe_3_O_4_ exhibited higher biocompatibility with SMMC-7721 and L02 cells as compared to uncoated ones and Ag NPs as well. Moreover, Ag coating could improve the in vitro and in vivo tumor therapeutic effect significantly. Besides, superparamagnetic Fe_3_O_4_@Ag (core-shell) and Fe_3_O_4_-PAA showed saturation magnetization (Ms) values of 75.1 and 82.4 emu/g, and SAR values of 76 and 87 W/g, respectively. Interestingly, the thin layer of Ag coating had no great influence in the reduction of MFH efficiency [191].

## 7. Discussion and Perspectives

The experiments conducted in the 1970s and 1980s provided a solid rationale and created a lot of enthusiasm for combining HT with RT. Clinical studies, however, failed to deliver the expected results, resulting in disappointment and loss of interest.

In retrospect, those studies were premature, as both treatment techniques were far from mature. Radiation therapy treatment planning was 2D, based on radiographs; routine clinical use of computed tomography (CT) and magnetic resonance imaging (MRI) was still in the future; dose was calculated at one or a few points, using measurement-based approaches; tissue inhomogeneities were often ignored; radiation fields were large, mostly rectangular with corner blocks and modulated using wedges and blocks; conformality was not high, so doses were limited.

On the hyperthermia side, the difficulties were much more severe. The physics of the problem, such as heat diffusion and convection by the bloodstream, made it very difficult to model the heating process. Heating devices were often unable to adequately heat the tumor, as exemplified by the RTOG study discussed in Section 5 [132].

In the 1990s and 2000s, RT experienced rapid technological progress: CT, MRI, and the explosion in computing power made possible truly 3D visualization of the patient’s internal anatomy; the accuracy and resolution of dose calculation algorithms increased sharply, with Monte Carlo algorithms likely to become available for routine clinical use in the near future; advances in linear accelerator design made possible the generation of highly conformal and complex dose distributions, such as selective boosting of tumor subvolumes (“dose painting”); quality assurance tools improved sharply, thus ensuring the accurate delivery of the intended treatment etc. In turn, these advances permitted the exploration of novel approaches, such as high dose per fraction hypofractionated treatments, as well as the development of radiobiological models to predict the response of tissues to radiation, as opposed to traditional observation-based approaches. The excitement generated by these advances further shifted attention away from HT, reducing the interest and funding available to improve heating devices, conduct studies etc.

For routine clinical use of HT, the following issues at a minimum need to be resolved: (a) accurate treatment planning, (b) heating devices and techniques that reliably and reproducibly deliver the intended treatment plan, and (c) quality assurance tools. Although several groups are working on these issues, significant problems remain, such as calculating the temperature distribution, modeling heat diffusion through tissues and heat convection through the bloodstream etc. [192,193,194,195,196]. Further, a well-designed and rigorous quality assurance protocol not only helps ensure that the treatment is delivered as intended, but also that, in case of treatment failure, the causes are identified.

MNPs enjoy certain characteristics that may address many of the problems associated with traditional HT:

(a) Heating takes place in situ, in contrast to traditional methods that heat from the outside in, thus avoiding problems such as diffusion and convection [20,134].
(b) The superparamagnetic nature of MNPs assures good hyperthermic efficacy even at low field amplitudes, thus allowing the use of light instrumentations for treatment delivery.
(c) The distribution of the MNPs can be determined using CT, MRI, SPECT, and PET, provided the MNPs are labeled with the appropriate radionuclide [138,151,167,197,198]. Alternatively, the magnetoacoustic properties of the MNPs can be used for localization [199,200,201]. This facilitates treatment planning, the modeling of the heating process and quality assurance.
(d) MNPs can act as contrast agents in MRI (theranostic action) [151,202].
(e) The MNPs can be used as temperature probes, thus providing non-invasive, real-time, 3D temperature distribution [203,204]. This is in contrast to standard methods of thermometry, such as optical fibers, that are both invasive and can measure the temperature only at a few points.
(f) In principle MNPs can act as self-regulated heat mediators, i.e., capable of avoiding overheating by switching off at a known temperature. This can be realized by exploiting materials whose Curie temperature is in the therapeutic range (40–50 °C), for example La_1−x_Sr_x_MnO_3_ [205].
(g) External magnetic fields can be used to direct the MNPs to the tumor (magnetic targeting) [206,207,208,209].
(h) Stimulation of the MNPs can be spatially varying, resulting in non-uniform temperature distributions by design [210,211], similar to “dose painting” in RT.

The above characteristics are a consequence of the magnetic nature of MNPs. In addition, MNPs enjoy advantages due to their small size and chemistry:

(i) Unlike macro or microscopic ferromagnetic implants, MNPs can be introduced in the tumor without surgery, which is feasible only for large and accessible tumors. In fact, one possible route of administration is by injection into the bloodstream [165,212,213,214,215].
(j) Since MNPs can be transported through the bloodstream, they may be used to treat small and surgically not addressable tumors, such as diffuse tumors and metastases.
(k) Their surface can be functionalized with specific targeting molecules able to “discover” the cancer cells and selectively attach to them, preserving the healthy tissues (chemical targeting) [151,216,217].
(l) They can take advantage of various physiological characteristics of tumors, such as the leaky and disorganized vasculature of the tumor and the Enhanced Permeation and Retention (EPR) effect, in order to accumulate in the tumor at higher concentrations [151,218,219].
(m) They can be used as drug carriers, to deliver and release specific drugs in situ after activation by an external magnetic field (coadjutant action) [220].

At present, many aspects related to the intratumoural accumulation and distribution of the MNPs remain to be addressed. Poor perfusion within the tumor core, the vascular barrier, the high interstitial pressure and the dense intracellular tumor matrix could limit nanoparticle delivery and their uniform distribution [221]. For systemic administration, which is the goal, opsonization, capture by the reticuloendothelial system, and removal through the filtering organs (kidneys, liver, spleen) are issues to be overcome. Further, the regulatory authorities require that the safety and efficacy of any new treatment be demonstrated before approval for clinical use. Thus, risk assessment studies must be carried out and quality assurance tools and procedures must be developed [222,223,224].

Challenges in exploiting the multifunctional potential of MNPs include structural design, biocompatibility, target specificity, optimal accumulation, dual magneto-photothermal mechanism and residence time in circulation. Furthermore, the production cost, cost effectiveness, quality control, standardization, and harmonization of regulations in different countries for the clinical use of MNPs need to be defined [223,225,226,227].

Considering the evolution of science and technology, it is probably a safe bet to assume that, sooner or later, these challenges will be overcome. MNPs will then be able to operate simultaneously as heat mediators, contrast agents, drug carriers etc. A multifunctional MNP with appropriate payloads could exploit the tumor vascularity to provide a unique opportunity to improve cancer therapy by integrating tumor imaging, radiotherapy, chemotherapy, immunotherapy, hyperthermia, and gene silencing therapy.

## 8. Conclusions

The combination of HT with RT results in a treatment that is more efficacious than the sum of HT and RT separately. Routine clinical adoption, however, has been hampered by practical and technical difficulties. In this work, we have reviewed the radiobiological evidence for combining HT with RT, and have described a new form of HT, namely, magnetic nanoparticle-mediated HT. We have discussed the potential of this technique to address the various issues related to traditional HT approaches, as well as the new possibilities that the multifunctional nature and capabilities of MNPs create.
